# The MQRG score: a novel prognostic tool for adrenocortical carcinoma patients based on mitochondrial quality

**DOI:** 10.3389/fendo.2024.1222281

**Published:** 2024-03-05

**Authors:** Tao Chen, Yifan Wang, Xue Chen, Wenbin Zheng, Weiquan Guo, Qi Liang, Jing Wang, Zhongbiao Chen, Yiwen Zhou, Lijia Xiao

**Affiliations:** ^1^ Shenzhen Key Laboratory of Viral Oncology, The Clinical Innovation & Research Centre, Shenzhen Hospital, Southern Medical University, Shenzhen, Guangdong, China; ^2^ The Third School of Clinical Medicine, Southern Medical University, Shenzhen, Guangdong, China; ^3^ Department of Neurology, Shenzhen Hospital, Southern Medical University, Shenzhen, Guangdong, China; ^4^ Department of Clinical Laboratory Medicine Center, Shenzhen Hospital, Southern Medical University, Shenzhen, Guangdong, China

**Keywords:** adrenocortical carcinoma, mitochondrial quality related gene (MQRG), MQRG score, tumor microenvironment, prognosis

## Abstract

**Objectives:**

Adrenal tumors are common, but adrenocortical carcinomas (ACCs) are a rare and challenging form of cancer to diagnose and manage.This study aimed to explore the critical role of mitochondrial quality in maintaining cellular function and the implications of the abnormal expression of mitochondrial metabolism-related proteins observed in ACC patients. We focused on identifying the connection between mitochondrial quality and the development of ACC at molecular and genomic levels.

**Methods:**

We compared mitochondrial quality-related genes (MQRGs) across ACC subtypes using overall survival (OS) and disease-free survival (DFS) as evaluation indicators. Furthermore, a novel MQRG score was developed to predict clinical prognosis and guide immunotherapy responses accurately.

**Results:**

The majority of MQRGs were upregulated in the ACC samples, correlating to poor prognosis. The MQRG score was confirmed as an independent prognostic factor for ACC, with the high-risk MQRG score group showing a significantly shorter overall survival period.

**Conclusions:**

Multilayer alterations in MQRGs are associated with patient prognosis and immune cell infiltration characteristics. This comprehensive analysis of MQRGs can contribute to a deeper understanding of potential differences in ACC patients' tumor microenvironment. This can influence clinical decision-making and advanced prognosis prediction, thereby offering new insights into personalized treatments in ACC.

## 1 Introduction

Adrenal tumors are prevalent, but adrenocortical carcinomas (ACCs) pose as rare and formidable cancer types, presenting challenges in both diagnosis and management. The spectrum of clinical presentations in adrenal tumors spans from adrenal cortical adenomas (ACAs) to more severe manifestations like ACC. Treatment modalities for adrenal tumors encompass surgical excision, radiotherapy, chemotherapy, and targeted therapy (1). However, effective therapeutic strategies for ACCs remain elusive. A significant hurdle in addressing adrenal tumors lies in their frequent manifestation of hormone activity, leading to clinical symptoms such as hypertension and weight gain in patients (2). Furthermore, the molecular mechanisms underlying ACCs and their malignant progression are intricate, and our current understanding falls short, posing challenges in devising effective therapeutic interventions.

Conducting genetic research is imperative for advancing our comprehension of adrenocortical carcinomas (ACCs) and identifying more efficacious clinical treatment modalities. A study indicates a close correlation between the occurrence of adrenal cortical tumors and adenosine monophosphate-activated protein kinase A, adrenocorticotropic hormone (ACTH), and the Wnt pathway (3). In pediatric ACC patients in southern Brazil, the frequently observed R337H mutation in the oligomerization domain of the p53 protein constitutes a prevalent germ line mutation, resulting in the substitution of arginine with histidine at codon 337 (4). Furthermore, sporadic ACCs have been reported to manifest gene rearrangements, loss of heterozygosity (LOH) at the 11p15.5 locus (loss of one of the two alleles of a gene), and abnormal imprinting at the 11p15.5 locus. This leads to diminished expression levels of p57 kip2 and H19, coupled with elevated expression levels of IGF2 mRNA (5, 6). Increased IGF2 expression levels correlate with more malignant phenotypes, and IGF2 overexpression is associated with an elevated risk of ACC recurrence. Despite the potential diagnostic value of IGF2 and MIB1, the quest for new molecular markers in the domain of diagnostic medicine remains an urgent priority.

Both genetic and proteomic data consistently reveal the frequent overexpression of the insulin-like growth factor 2 (IGF2)-H19 locus in adrenocortical carcinomas (ACCs). Studies underscore a robust correlation between IGF2 overexpression and the expression of miR-483-3p and miR-483-5p, both originating from the IGF2 host (7). Moreover, ACCs exhibit alterations in various proteins associated with mitochondrial metabolism (7). Mitochondria play a pivotal role in supplying cellular energy and regulating diverse cellular processes, including redox balance, carcinogenic signal transduction, innate immunity, and cell apoptosis (8). They are indispensable for maintaining tissue homeostasis and programmed cell death. Mitochondrial quality and quantity are governed by mitochondrial dynamics (fusion and fission), mitochondrial autophagy (elimination of damaged mitochondria through autophagy), and mitochondrial biogenesis (9). Imbalances in mitochondrial functions are closely linked to the development of various human diseases, including tumors.

Maintaining mitochondrial quality is indispensable for normal cellular function, and compromised mitochondrial function may contribute to the development of various human diseases, including cancer (10). The energetic demands of proliferating cancer cells are primarily met through the mitochondrial oxidative phosphorylation system. Disruptions in energy production and alterations in mitochondrial oxidative metabolism, influencing mitochondrial quality, can consequently facilitate the initiation and progression of tumors. Additionally, changes in mitochondrial DNA, housing genes essential for oxidative phosphorylation, can impact both mitochondrial quality and cellular function, increasing susceptibility to cancer. Associations have been established between mitochondrial quality and the incidence of various cancer types, including liver cancer, breast cancer, colon cancer, and prostate cancer (11, 12). Therefore, a comprehensive understanding of the mechanisms governing mitochondrial quality control in tumor pathogenesis is paramount for devising innovative cancer treatment strategies.

Mitochondrial Quality Control (MQC) forms an intricate biological network that, through mechanisms like autophagy, fusion, fission, and dynamics regulation, contributes to maintaining normal mitochondrial function. Numerous studies have underscored the crucial role of MQC in various diseases. For example, Cai et al. delved into the impact of MQC in diabetic cardiomyopathy, revealing that disrupted MQC accelerates disease progression by impairing mitochondrial function (13). Similarly, Chang et al. explored therapeutic strategies for ischemic myocardial disease, highlighting MQC’s protective role against myocardial ischemic damage (14). Picca investigated how defective MQC mechanisms contribute to cardiac aging, exploring potential molecular pathways and drug treatments (15). Zhong studied the relationship between regulatory factors and AML biological characteristics, identifying SRSF10 as a potential target for AML treatment and biomarker (16). Finally, Zou demonstrated how empagliflozin enhances mitochondrial homeostasis, protects cardiac microvasculature, and alleviates myocardial ischemia/reperfusion injury (17). Aberrations in the structure and function of mitochondria observed in various tumor types lead to disruptions in quality control mechanisms, abnormalities in oxidative metabolism, and mitochondrial fission. Studies also indicate that mutations in genes involved in metabolic pathways lead to mitochondrial metabolic disorders, thereby promoting cancer development. A comprehensive understanding of the interplay between mitochondrial quality control and metabolic regulatory mechanisms in cancer development holds promise for developing innovative cancer treatment strategies.

Addressing various diseases arising from mitochondrial dysfunction has prompted intensive research into identifying actively present genes or proteins within mitochondria. In this context, Mitochondrial Quality-Related Genes (MQRGs), including PPARGC1A, PPARA, PPARG, NRF1, NFE2L2, TFAM, ESRRA, MFN1, MFN2, OPA1, MFF, FIS1, MIEF2, MIEF1, PINK1, PARK2, SQSTM1, MAP1LC3A, MAP1LC3B, MAP1LC3C, originated directly from the comprehensive mammalian mitochondrial protein inventory provided in MitoCarta 3.0, stand out as a promising research tool (18). Detailed insights into the methodology employed can be found in cited manuscript. MQRGs play a critical role in regulating mitochondrial function, and understanding their diverse expression patterns and their impact on organismal function is crucial. In this study, we provide a systematic description of MQRG expression in adrenocortical carcinoma (ACC) and assess their expression patterns in the TCGA-ACC dataset. Utilizing unsupervised clustering, we identified two distinct classes of ACC samples with significantly different overall survival rates and identified differentially expressed genes between these classes. Building upon this, we introduced a scoring system, the MQRG Gene-Mediated Scoring System (MQRG Score), which categorizes samples into high and low score groups associated with markedly different overall survival. Our model underwent validation using a validation set. Subsequently, we conducted a comprehensive analysis to explore the correlation between the MQRG score and various factors, including clinical features, levels of immune infiltration, immune characteristics, expression of immune checkpoints, and immune efficacy. The results underscore the multi-layered changes in MQRGs linked to patient prognosis and immune cell infiltration characteristics. This study aims to comprehensively depict the dysregulation of MQRGs in tumors, explore variations in MQRG regulation under different patterns, and elucidate the potential molecular mechanisms and drug responses of MQRGs under various regulatory modes.

## 2 Method

### 2.1 Data acquisition and processing

In this research, we assessed MQRGs through a comprehensive literature search. The selection of these genes was based on their established associations with potential biological mechanisms of the studied phenotype, as reported in previous studies (1). We obtained mRNA expression profile data and sample CNV information from the XENA database (https://xenabrowser.net/datapages). Clinical information was obtained using the R package cgdsr, and mutation data were acquired using the R package TCGA-biolinks. For the GSE10927 dataset, we downloaded expression data and sample survival information from the GEO database (https://www.ncbi.nlm.nih.gov/geo/) and summarized the survival information for the samples. Additionally, we obtained immunotherapy data from IMvigor210CoreBiologies.

### 2.2 Information on the mutation and copy number variation, expression, and chromosomal location of MQRGs

Initially, we employed the Circos (R package) to visually represent the specific distribution of MQRGs on the chromosomal level. Subsequently, we conducted Wilcoxon tests to analyze the expression differences of MQRGs between normal and tumor samples, using adrenal tissue from GTEx as the control group (n=128). Utilizing the maftools (R package), we showcased the mutation landscape of MQRGs, offering a comprehensive visualization of the global mutation status of these genes. Likewise, we presented the copy number variations of MQRGs in the overall tumor samples. Following this, we conducted survival analysis of MQRGs expression and overall survival, exploring the correlation between MQRGs expression and overall survival using One-way cox regression analysis. Additionally, we utilized CIBERSORT to evaluate immune infiltration in tumor samples and analyzed the correlation between MQRGs expression and the abundance of immune cells (Pearson correlation).

### 2.3 Identifying subtypes based on MQRGs

To identify subtypes based on the expression of MQRGs, we performed Consensus Clustering analysis using the ConsensusClusterPlus package. We opted for the hc distance and Pearson clustering methods, conducting 1000 repetitions to ensure the stability of the classification. This not only addressed the survival situation of different subtype samples but also enabled accurate classification of tumor samples based on the similarity in the expression levels of MQRGs. This, in turn, contributes to a more profound understanding of the potential mechanisms and treatment options for each subgroup.

### 2.4 Gene set variation analysis

To investigate the expression pattern differences in biological processes among different sample types, we conducted Gene Set Variation Analysis (GSVA) enrichment analysis using the R package GSVA. This analysis utilized RNA-seq data from TCGA-ACC. GSVA, a non-parametric and unsupervised method, is primarily employed to estimate changes in the activity of pathways and biological processes within samples. We retrieved the gene set h.all.v7.4.symbols.gmt from the MSigDB database (https://www.gsea-msigdb.org) and employed it for GSVA analysis. This enabled us to compare the enriched biological processes in different sample types.

### 2.5 Identifying MQRG-related differential genes

Building upon the identified subtypes, we utilized limma to pinpoint genes exhibiting significant differential expression between subtypes. The analysis was conducted using TCGA-ACC RNA-seq data. We applied filtering criteria of |log2FC| > log2 (5) and an adjusted P-value< 0.05. After this, we subjected these differentially expressed genes to Gene Ontology (GO) and Kyoto Encyclopedia of Genes and Genomes (KEGG) enrichment analysis, identifying significantly enriched biological processes and related pathways (P< 0.05). Following that, we employed the ConsensusClusterPlus package for consistency clustering analysis, incorporating hc distance and Pearson clustering methods. Through 1000 repetitions, we ensured the stability of the classification and addressed the survival situation of different categories. This comprehensive method enhances our understanding of molecular processes and pathways associated with different subtypes, potentially revealing novel insights into therapeutic strategies for each subtype.

### 2.6 MQRG score prognostic assessment system development and efficacy analysis

To establish a reliable prognostic assessment system, we amalgamated the differentially expressed genes identified in the previous steps and conducted univariate Cox regression analysis to identify genes significantly associated with overall survival (P< 0.001). Utilizing these prognostically relevant genes, we constructed a prognostic assessment system based on MQRG scores using Principal Component Analysis (PCA) for survival prediction. The formula for calculating MQRG scores is as indicated:


$$\text{MQRG}-\text{score}=\sum (PC1_{i}+PC{2i}_{)}$$

We obtained the PC and PC2…PCn for each sample using a PCA analysis of the expression spectrum of the aforementioned 10 genes. To validate the scoring system, we applied the same formula in the validation set, using the median as the grouping threshold for high and low MQRG score samples. Furthermore, we assessed the predictive capability of the scoring system using Kaplan-Meier survival analysis and receiver operating characteristic curve (ROC curves). To explore the potential of MQRG scores as independent prognostic factors, we conducted univariate and multivariate Cox risk analyses on both the training and validation sets. Additionally, we analyzed the distribution differences of clinical features among different MQRG score groups. Through these comprehensive analyses, our goal is to develop a robust prognostic scoring system with practical applications in clinical settings.

### 2.7 Differences in mutations and CNV between high and low scoring groups

To delve deeper into the genetic differences between high and low MQRG score groups, we employed GISTIC2 analysis on the GenePattern website to examine copy number variation (CNV) data in somatic cells. Based on the categorization of the aforementioned samples, we identified regions significantly amplified or deleted in the CNV data within each group. Furthermore, we assessed the frequency of mutated genes in high and low MQRG score groups to ascertain if there are notable differences. Through a comprehensive investigation of these genetic disparities, our objective is to gain a more profound understanding of potential biomarkers and potential therapeutic targets associated with each group.

### 2.8 Immunotherapy analysis

To validate the clinical applicability of the MQRG score prognostic assessment system, we applied it to an independent immune dataset, specifically the IMvigor210CoreBiologies dataset. By comparing the distribution of MQRG scores between responders and non-responders to immunotherapy, we assessed the predictive capacity of our scoring system in the immune dataset. We also incorporated the immune signature gene set, calculating enrichment scores for immune signatures across all tumor samples (19). Our aim is to showcase the universality and robustness of our scoring system across diverse cancer patient populations. In essence, we believe the successful validation of our scoring system in the immune dataset provides additional evidence supporting its potential clinical utility.

### 2.9 Statistical analysis

Statistical analyses were conducted using R software v4 and GraphPad Prism v9. In statistical plots, ns denotes P >= 0.05, * represents P< 0.05, ** indicates P< 0.01, *** signifies P< 0.001, and **** is used for P< 0.0001.

## 3 Results

### 3.1 Mutation and copy number variation of MQRGs, expression and chromosome location information

**Figure 1:**
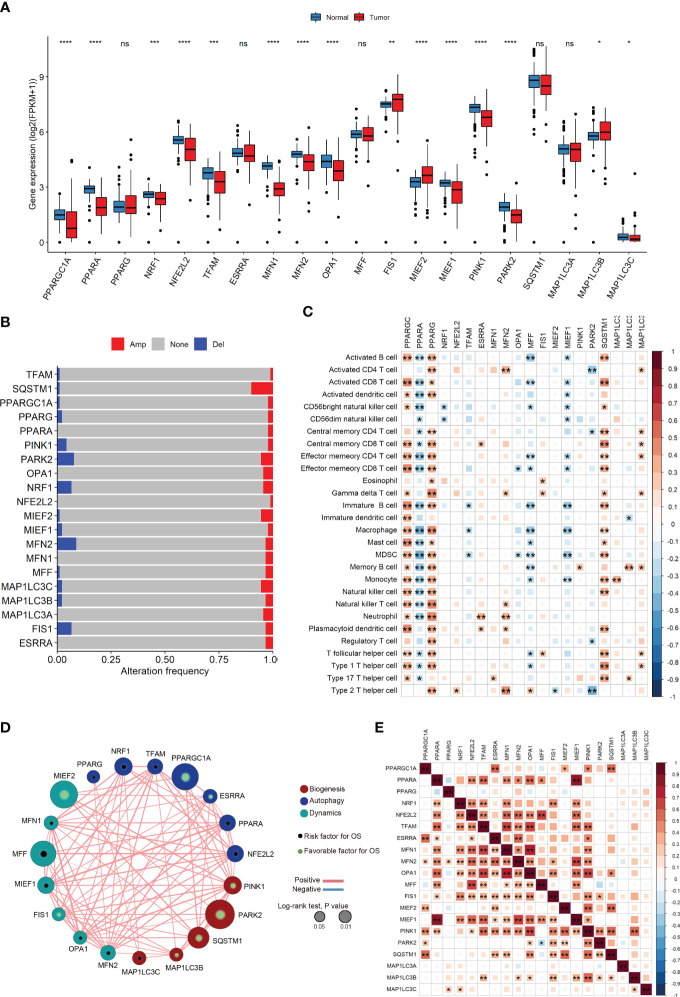
**(A)** The Mitochondrial Quality-Related Genes (MQRGs) expression in tumor and normal tissues. Using the wilcoxon-test to analyze the differences of MQRGs expression between tumor and normal tissues. **(B)** The maftools package was used for MQRGs on the copy number variation of MQRGs in the tumor sample. Red, Amplification (Amp). Blue, Deletion (Del). Gray, No mutation (None). **(C)** CIBERSORT was used to calculate the immune cell abundance of tumor samples, and the correlation between immune cell abundance and MQRGs expression was performed using the psych package in conjunction with MQRGs expression. **(D)** One-way cox regression analysis was used for the analyze of the correlation between MQRGs and overall survival (OS). The node size reflects the significance associated with prognosis, with node color indicating the gene category. Green and black markers within the nodes denote positive and negative correlations with prognosis, respectively. Edge color is represented in red and blue, signifying positive and negative correlations, respectively. **(E) **The expression levels between MQRGs. Significant positive correlations are observed in the expression levels among the majority of MQRGs. *P < 0.05, **P < 0.01, ***P < 0.001 and ****P < 0.0001, Ns denotes P >= 0.05.

We observed substantial differences in the expression levels of most MQRG between tumor and normal tissues (P<0.05, **Figure 1A**). Our investigation into MQRG mutations and copy number variations revealed amplifications and deletions predominantly in ACC (**Figure 1B**). Furthermore, a noteworthy correlation between MQRG expression levels and the abundance of immune cells was observed (**Figure 1C**). To delve deeper, we evaluated the relationship between MQRG expression and overall survival (OS), identifying seven MQRG significantly associated with overall survival. Specifically, NRF1 and MFF exhibited a negative correlation with overall survival, while PARK2, MIEF2, PPARGC1A, SQSTM1, and PINK1 displayed a positive correlation (P<0.05, **Figure 1D**). Furthermore, we investigated the expression levels among MQRGs. A substantial number of MQRGs exhibit a pronounced and statistically significant positive correlation in their expression patterns (**Figure 1E**). These findings suggest that MQRG expression could serve as a valuable indicator for both ACC diagnosis and prognosis.

### 3.2 Identification of MQRG-related ACC subtypes

**Figure 2:**
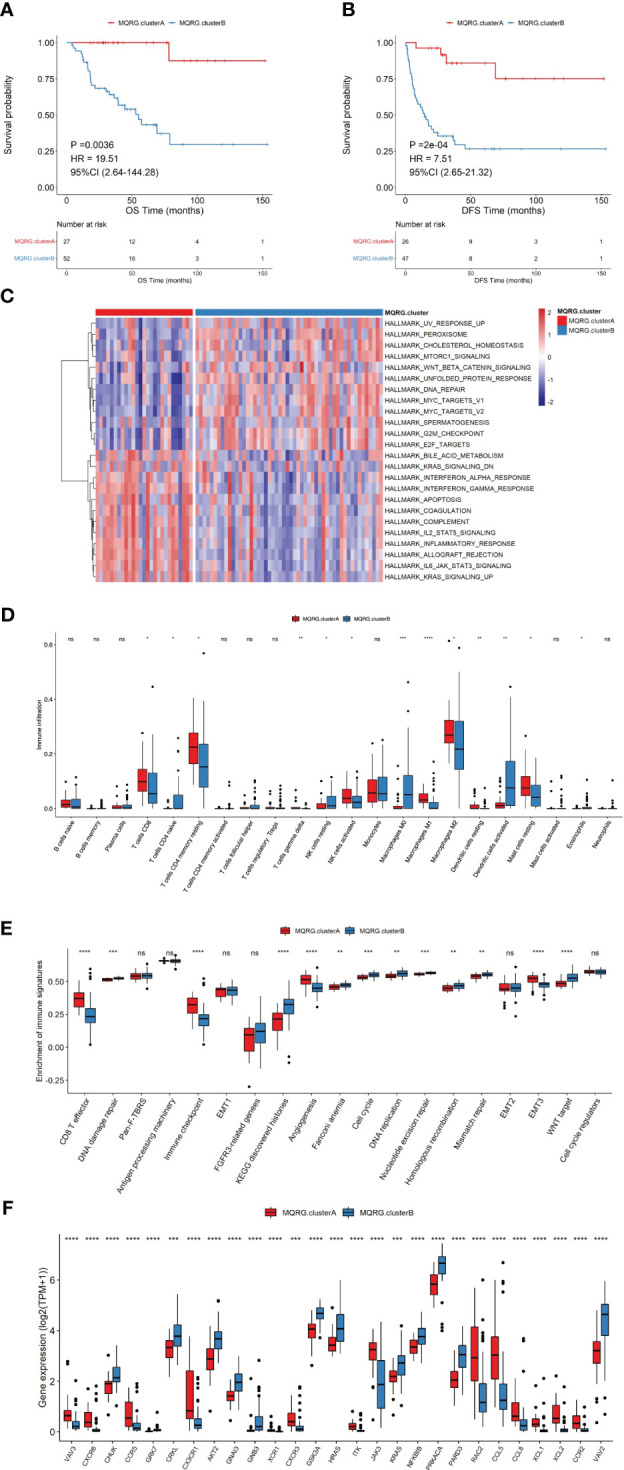
**(A, B)** Two subtypes were identified using consensus clustering based on Mitochondrial Quality-Related Genes (MQRGs) expression (MQRG.clusterA, MQRG.clusterB). The survival analysis showed significant differences in overall survival (OS) and disease free survival (DFS) between these two subtypes, with MQRG.clusterA showing significantly better OS and DFS. **(C)** GSVA analysis indicated a total of 24 significantly different pathways between the two subtypes. Heatmap depicting GSVA values for various Hallmark pathways after scaling. The color intensity of the scale bar illustrates the relative activity levels of each pathway, with darker colors indicating higher activity or greater inhibition compared to other pathways. **(D)** To assess the differences in immune cell abundance between samples belonging to different MQRG clusters, we employed the Wilcoxon test. Significant variations in the abundance of several immune cell types, including Macrophages M0, Macrophages M1, and T cells gamma delta, were observed between most pairs of samples from distinct MQRG clusters. **(E) **The immune signature gene set was used to calculate the 19 immune signature enrichment profiles for all tumor samples using single-sample gene set enrichment analysis (ssgsea). the MQRG.clusterA group exhibited significantly higher enrichment scores, particularly in CD8 T effector, immune checkpoint, and angiogenesis immune signatures. **(F)** The wilcoxon-test was used to resolve the differences in the expression of immune-chemokines between the two subtypes, and the expression of 27 highly significant immune-chemokine genes was shown. *P < 0.05, **P < 0.01, ***P < 0.001 and ****P < 0.0001, Ns denotes P >= 0.05.

**Figure 3:**
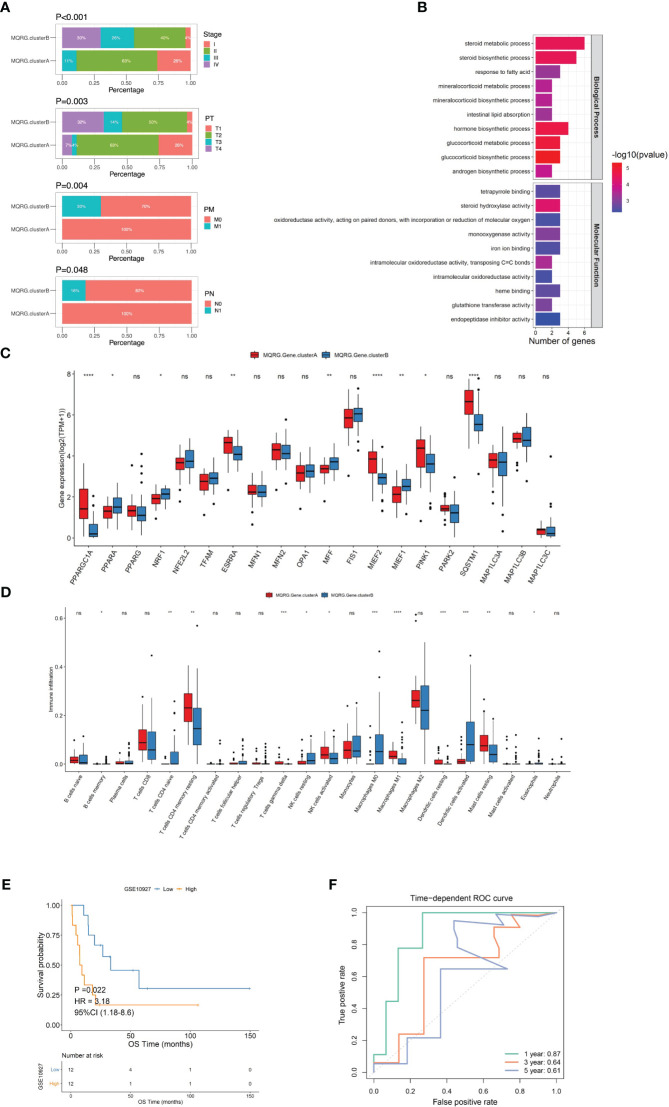
**(A)** We utilized the chi-square test to analyze the distribution of clinical feature categories among samples of different clusters. It is evident from our analysis that there are significant differences in the distribution of Stage, PT, PM, and PN among samples belonging to different categories. Pathological Tumor (PT), Pathological Metastasis (PM), Pathological Node (PN). **(B) **To construct the scoring system (MQRG score), we identified 35 differential genes from two subtypes. The clusterProfiler was used to perform functional enrichment analysis of the differential genes. **(C)** The box plots showed some MQRGs expression was significantly different in these two subtypes. **(D)** Wilcoxon test was employed to examine the differences in immune cell abundance between MQRG gene clusters. Our analysis revealed significant differences in the abundance of certain immune cell subtypes between the two subsets. Notably, immune cells such as Macrophages M0, M1, and Dendritic cells resting exhibited substantial variations. **(E, F)** A one-way cox regression model was used to screen genes that were significantly associated with OS using a threshold of P<0.001 as the trait genes, we obtained a total of 10 differential genes significantly associated with OS (FSCN1, NDRG4, SLC30A2, EMB, PRLR, CYP27A1, CSDC2, GSTA1, C3, GSTA2). The median MQRG score was utilized to stratify the samples into high and low-scoring groups. Subsequently, Kaplan-Meier (KM) curves were generated, unveiling significant differences in overall survival (OS) within the validation set. The receiver operating characteristic curve (ROC curves) also show that the model has good predictive performance, with an area under the curve (AUC) of 0.85 for 5-year survival in the training set and 0.87 for 1-year survival in the validation set. MQRG, Mitochondrial Quality-Related Gene. *P < 0.05, **P < 0.01, ***P < 0.001 and ****P < 0.0001, Ns denotes P >= 0.05.

Utilizing MQRG expression (PPARGC1A, PPARA, PPARG, NRF1, NFE2L2, TFAM, ESRRA, MFN1, MFN2, OPA1, MFF, FIS1, MIEF2, MIEF1, PINK1, PARK2, SQSTM1, MAP1LC3A, MAP1LC3B, MAP1LC3C), we categorized ACC samples into two clusters (MQRG low, Cluster A, and high, Cluster B) and examined the significant differences in gene expression between these groups. Survival analysis unveiled noteworthy disparities in both overall survival (OS) and disease-free survival (DFS) between the clusters. Notably, samples expressing lower levels of MQRG exhibited significantly superior OS and DFS (**Figures 2A, B**). To delve into the alterations in biological pathways within MQRG-related subtypes, we conducted GSVA analysis, identifying 24 pathways with significant differences between the two groups (P<0.05). The MQRG low-expression group displayed notable enrichment in pathways related to interferon-alpha response, interferon-gamma response, and apoptosis. In contrast, the MQRG high-expression group showed significant enrichment in pathways associated with DNA repair, MYC target V1, and MYC target V2 (**Figure 2C**). Within the two MQRG-related subtypes, significant variations in the abundance of immune cells, including macrophages M0, macrophages M1, and T cells gamma delta, were observed (**Figure 2D**). Calculating the enrichment scores of immune features revealed significantly higher scores in the MQRG low-expression group for features such as CD8 T effector, immune checkpoints, and angiogenesis (**Figure 2E**). Furthermore, we also employed the Wilcoxon-test to analyze the expression differences of immune chemokine factors between two sample categories. The expression levels of 27 immune chemokine factor genes were significantly changed. It is evident that genes such as VAV3, CXCR6, and CCR5 show significantly higher expression in MQRG.clusterA (**Figure 2F**). Chi-square tests highlighted significant differences in the distribution of clinical features across distinct sample clusters, encompassing stage, Pathological Tumor (PT), Pathological Metastasis (PM) and Pathological Node (PN) (**Figure 3A**). These findings suggest a potential role for MQRG in regulating immune response and DNA repair pathways, thereby impacting tumor development and clinical prognosis.

### 3.3 Construction of the MQRG scoring system

To delve deeper into the functional roles of the identified MQRG subtypes, we pinpointed 35 differentially expressed genes (S1PR3, FSCN1, NDRG4, NFATC4, SLC30A2, UGCG, COL14A1, ASB4, EMB, PRLR, SLPI, CYP27A1, FABP3, MC2R, CSDC2, QPCT, LDLR, HSPB7, G0S2, PEG10, ALPL, NPTX1, SNRPN, GSTA1, PDE2A, PCP4, NDN, CYP21A2, CYP17A1, BIRC7, HSD3B2, C3, KLK1, GSTA2, SPRR1A), observing enrichment in steroid metabolic processes, steroid biosynthetic processes, and responses to fatty acids (**Figure 3B**). We utilized the Wilcoxon test to examine the significant differences in the expression of certain MQRG members between MQRG.gene.clusterA and MQRG.gene.clusterB, confirming the presence of substantial differences in expression (**Figure 3C**). Grouping the subtypes into MQRG.gene.clusterA and MQRG.gene.clusterB based on gene expression values revealed significant disparities in immune cell populations (especially macrophages M0, M1, and resting dendritic cells) (**Figure 3D**). The identification of the 10 trait genes was conducted through a univariate Cox regression model, with a significance threshold set at P<0.001. Genes exhibiting a significant correlation with Overall Survival (OS) were selectively chosen as candidate trait genes. Consequently, a total of 10 genes (FSCN1, NDRG4, SLC30A2, EMB, PRLR, CYP27A1, CSDC2, GSTA1, C3, GSTA2) were identified as being significantly associated with OS. Subsequent survival analysis highlighted significant differences in OS and DFS between high and low MQRG scoring groups, with an area under the curve (AUC) of 0.87 for 1-year survival (**Figures 3E, F**).

**Figure 4:**
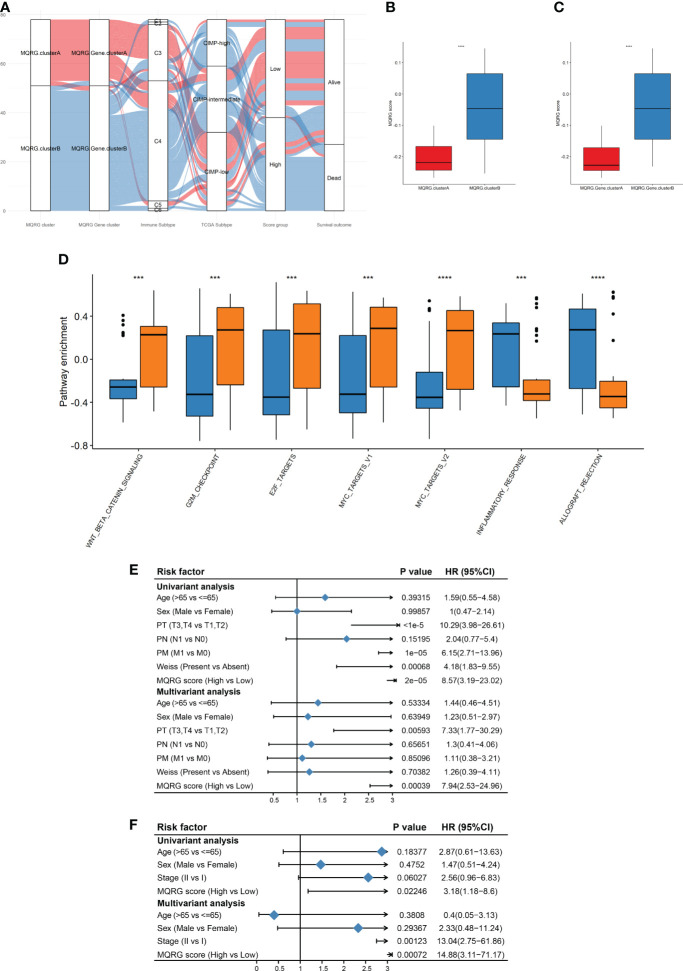
**(A)** Alluvial diagram was used for their corresponding with immune categories, TCGA categories, and overall survival (OS). Immune subtypes: wound healing (C1), IFN-g dominant(C2), inflammatory(C3), lymphocyte depleted(C4), immunologically quiet(C5), and TGF-b dominant(C6). TCGA subtypes: CpG island methylator phenotype-high (CIMP-High), CpG island methylator phenotype-intermediated (CIMP- intermediated), CpG island methylator phenotype-low (CIMP-low). **(B-C)** By comparing the MQRG scores between different subtypes, we found that the MQRG scores were significantly different among the samples of different categories, with clusterB having a significantly higher MQRG score. **(D)** Differences in hallmark pathway enrichment scores between MQRG score groups were resolved using the wilcoxon-test. **(E, F)** To explore whether the MQRG score could be used as an independent prognostic factor, we evaluated the MQRG score in the training and validation sets by combining single-factor cox and multi-factor cox regression analyses, respectively. The TNM staging system, a universally recognized method for malignancy staging, categorizes primary tumors (T), lymph node involvement (N), and metastasis (M). Tumor size and extent, lymph node status, and the presence of distant metastasis are respectively represented by T stages (T0, T1-T4, Tis, Tx), N stages (N0, N1-N3, Nx), and M stages (M0, M1). Pathological Tumor (PT), Pathological Metastasis (PM), Pathological Node (PN). *P < 0.05, **P < 0.01, ***P < 0.001 and ****P < 0.0001, Ns denotes P >= 0.05.

Utilizing an Alluvial diagram, we illustrated the intricate relationship between MQRG scoring, immune categories, TCGA categories, and OS (**Figure 4A**). Immune subtypes and TCGA subtypes could be found in cited manuscript (20). Noteworthy differences in MQRG scoring emerged across samples in different categories, with clusterB exhibiting significantly higher MQRG scores (**Figures 4B, C**). Additionally, a substantial correlation was unveiled between MQRG scoring and hallmark pathways, particularly the WNT β-catenin signaling pathway, G2M checkpoint, and E2F targets (**Figure 4D**). In order to further explore whether the MQRG score could serve as an independent prognostic factor, we conducted joint univariate and multivariate Cox regression analyses on the training and validation sets. In the training set (including Age, Sex, PT, PN, PM, Weiss, and MQRG score), univariate Cox regression analysis revealed significant associations of PT, PM, Weiss, and MQRG score with overall survival (OS). Similarly, in the multivariate Cox regression analysis with these features, PT and MQRG score remained significantly associated with OS, indicating that in the training set, the MQRG score can function as an independent prognostic factor (**Figure 4E**). Likewise, in the validation set (including Age, Sex, Stage, and MQRG score), univariate Cox regression analysis showed a significant association between MQRG score and OS. Moreover, the multivariate Cox regression analysis, incorporating the aforementioned four features, revealed a sustained significant association between MQRG score and OS, demonstrating the independent prognostic capability of the MQRG score in the validation set (**Figure 4F**).

### 3.4 Evaluation of immunotherapy efficacy with MQRG score

**Figure 5:**
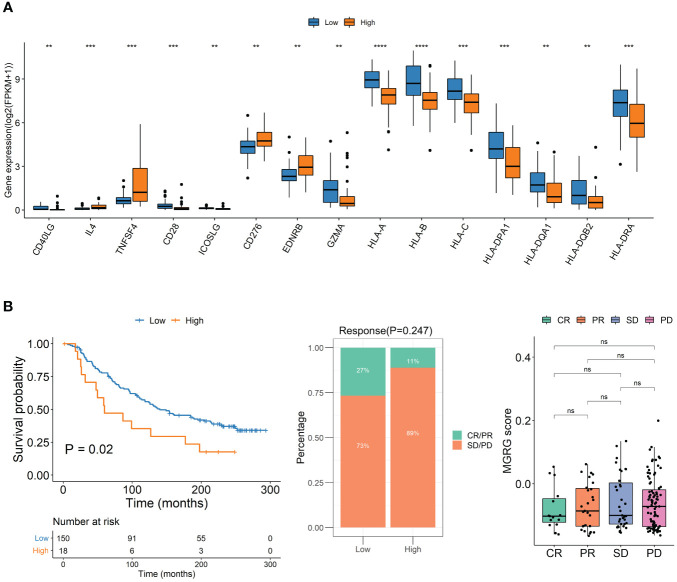
**(A)** Immune Checkpoint Gene Expression in High and Low MQRG Score Groups. Wilcoxon test identified 15 genes with significantly lower expression in high MQRG score group. **(B)** To study the effect of immunotherapy in high and low scoring groups, the KM survival curve illustrates overall survival outcomes, while the distribution of immunotherapy response categories in high and low MQRG score groups is depicted. Additionally, boxplots highlight MQRG score variations among different efficacy groups. These findings underscore the robust predictive capacity of the MQRG model in analyzing immunotherapy data, with a particular emphasis on predicting overall survival outcomes. CR, Complete Response. PR, Partial Response. PD, Progressive Disease. SD, Stable Disease. MQRG, Mitochondrial Quality-Related Gene. CR, Complete Response. PR, Partial Response. PD, Progressive Disease. SD, Stable Disease. MQRG, Mitochondrial Quality-Related Gene. *P < 0.05, **P < 0.01, ***P < 0.001 and ****P < 0.0001, Ns denotes P >= 0.05.

To explore differences between high and low MQRG scoring subgroups, we analyzed immune checkpoint genes. The results unveiled that the subgroup with higher MQRG scores exhibited significantly lower gene expression of immune checkpoint genes (**Figure 5A**). These findings reinforce the potential crucial role of MQRG gene in regulating the immune response and its potential as a valuable prognostic marker for ACC patients. To assess the impact of immunotherapy on high and low MQRG scoring subgroups, we delved into the IMvigor210CoreBiologies dataset. Initially computing MQRG scores, we subsequently categorized samples into two groups based on these scores. Throughout the analysis, no significant differences were observed between the complete response (CR)/partial response (PR) group and the progressive disease (PD) group. Moreover, within the high and low MQRG scoring groups, no notable changes were noted in the distribution of immune response categories. However, survival analysis pointed to a significantly poorer prognosis in the high-scoring group compared to the low-scoring group (**Figure 5B**). These findings underscore that this model serves as a reliable prognostic indicator for overall survival in immunotherapy data.

## 4 Discussion

Adrenocortical carcinoma (ACC) is a complex and heterogeneous disease, with its underlying mechanisms and treatment options still being inadequately understood. The identification of novel pathogenic mechanisms and treatment targets is indispensable for formulating more efficacious management and treatment approaches for ACC. Despite advancements, there remains an insufficient comprehension of the pathways involving MQRGs, emphasizing the need to explore new pathogenic mechanisms and treatment targets. Such research plays a pivotal role in advancing the development of more effective strategies for the management and treatment of ACC.

The pivotal role of mitochondria in the survival and growth of cancer cells cannot be overstated. Additionally, ensuring mitochondrial quality control (MQC) is indispensable for cancer cells, and the engagement of mitochondrial dynamics and the mitochondrial autophagy pathway in this process is crucial for developing effective cancer treatments targeting specific aspects of mitochondrial dynamics and autophagy (21). The significance of abnormal mitochondrial metabolism in adrenocortical carcinoma (ACC) has garnered attention, with research highlighting that energy metabolism stands out as a notable hallmark of the ACC phenotype. Mitochondrial metabolism is critical for normal cells and is governed by MQC (22). However, the disruption of MQC may contribute to the pathogenesis of ACC. As more information on abnormal mitochondrial metabolism surfaces, it opens the doors to novel and more effective treatments for ACC.

Several studies propose that mitochondrial quality control (MQC) holds promise as a crucial area in cancer treatment. Mitochondrial autophagy, a pivotal MQC mechanism, efficiently eliminates damaged mitochondria, enhancing overall mitochondrial function. Enhancing MQC function may lead to the suppression of tumor growth and heightened sensitivity to chemotherapy or radiotherapy (23). Conversely, compromising MQC may induce mitochondrial dysfunction, initiating tumor development. In cancer, mitochondrial autophagy experiences impairment, affecting mitochondrial autophagy receptors and adapters, including PINK1, Parkin, BNIP3, BNIP3L/NIX, and p62/SQSTM1, along with associated signaling pathways (24). Hence, targeting the MQC pathway emerges as a promising strategy for managing adrenal tumors. MitoCarta3.0 serves as an updated and comprehensive resource, offering researchers a repository of mitochondrial proteins. This repository is accompanied by detailed annotations, providing in-depth descriptions of protein functions, expression patterns, and subcellular localization, facilitating the comprehensive characterization of these proteins (18). In our study, we’ve demonstrated the pivotal role of Mitochondrial Quality-Related Genes (MQRGs) in regulating mitochondrial function and their potential to differentiate ACC patients. The significant expression differences of most MQRGs between tumor and normal tissues, along with frequent observations of copy number variations involving amplifications and deletions, underscore their relevance. Additionally, the positive/negative correlation between MQRG expression and immune cell abundance, along with the substantial positive correlations among most MQRGs, suggests that abnormal MQRG expression may contribute significantly to the development of ACC.

Tumor classification stands as a pivotal method for determining the malignancy and heterogeneity of tumors. The personalized classification of tumors based on individual patient characteristics can significantly assist in clinical diagnosis, treatment planning, and prognosis assessment. In our study, we conducted clustering analysis utilizing the expression profiles of Mitochondrial Quality-Related Genes (MQRGs). Survival analysis revealed noteworthy differences in both overall survival (OS) and disease-free survival (DFS) between two distinct sample clusters, with the MQRGs low-expression group exhibiting significantly better OS and DFS. Consequently, the characteristic high expression of MQRGs serves as an indicator of aberrant changes in carcinogenic pathways. Furthermore, we observed significant variations in immune cell abundance. The higher expression of MQRGs correlated with lower enrichment scores for immune features and the expression of 27 immune chemokine factor genes, signifying the presence of a tumor microenvironment characterized by the development of chronic inflammation and immune suppression. These findings offer crucial insights into understanding the potential mechanisms of mitochondrial function in adrenocortical carcinoma (ACC) and lay the groundwork for developing novel treatment strategies aimed at improving patient prognosis. However, the correlation analysis presented in the manuscript highlights the complexity of the relationship between MQRGs and ACC patient survival. It is crucial to clarify that the correlation does not imply causation, and the impact of individual MQRGs on patient outcomes may vary. These findings underscore the nuanced role of MQRGs in ACC, and further analyses are needed to elucidate the specific mechanisms underlying these associations.

To further reveal the functional roles of Mitochondrial Quality-Related Genes (MQRGs), we identified 35 differentially expressed genes encoding proteins. Employing a univariate Cox regression model, we screened for genes significantly associated with overall survival (OS) to select feature genes. This analysis pinpointed a total of 10 differentially expressed genes significantly correlated with OS. Following this, we computed MQRG scores in both the training and validation sets using a scoring system formula. Samples were subsequently categorized into high and low-score groups based on the median MQRG score. Our MQRG score model emerges as a valuable prognostic factor for patients with adrenal tumors. It encapsulates the overall quality of mitochondrial function and structure in the context of adrenocortical carcinoma (ACC), elucidating its connection with patient prognosis. This study sheds light on the role of mitochondrial quality control (MQC) in tumor initiation and development, offering crucial insights for the development of novel therapies for ACC.

The MQRG score proves valuable in classifying patients with adrenocortical carcinoma (ACC) and gaining insights into the patients’ pathological conditions and tumor microenvironment characteristics. In our pursuit of enhancing personalized treatment plans and prognosis assessments for ACC patients, we assessed the treatment sensitivity of patients with varying MQRG scores. Utilizing univariate and multivariate Cox regression analyses, we evaluated MQRG scores in both the training and validation sets. Significantly, we discovered a correlation between MQRG scores and overall survival (OS). Demonstrating independence as a prognostic factor, the MQRG score exhibited substantial differences in the expression of immune checkpoint genes between high and low-score groups. Notably, the group with high MQRG scores displayed significantly lower expression of immune checkpoint genes, suggesting the potential of our model as a prognostic tool for immune therapy. This model can be a valuable asset for clinicians in better predicting patient prognosis.

In-depth research is necessary to comprehensively grasp the potential mechanisms linking mitochondrial quality control (MQC) to the occurrence of adrenal tumors. These discoveries hold crucial clinical implications, offering valuable insights for the development of novel therapies for adrenocortical carcinoma (ACC) and setting the stage for more personalized treatments. This, in turn, has the potential to significantly enhance patient prognosis.

## 5 Conclusion

In summary, our investigation revealed the aberrant expression of proteins related to mitochondrial metabolism in patients with adrenocortical carcinoma (ACC), shedding light on the connection between mitochondrial quality and the development of ACC. Additionally, we clarified the correlation between MQRG expression and significant differences in overall survival (OS) and disease-free survival (DFS) across different ACC subtypes. Moreover, we introduced a novel MQRG score designed to assist in clinical prognosis and evaluate the response to immune therapy. This MQRG score was validated as an independent prognostic factor for ACC.

## Data availability statement

The raw data supporting the conclusions of this article will be made available by the authors, without undue reservation.

## Author contributions

LX and YZ were responsible for the comprehensive study design, paper revision, and submission. TC, YW and XC contributed to data extraction and performed statistical analysis. WZ, WG, QL, WJ and ZC contributed to article search. All authors contributed to the article and approved the submitted version.

## References

[1] Shah MH Goldner WS Benson AB Bergsland E Blaszkowsky LS Brock P. Neuroendocrine and adrenal tumors, version 2.2021, nccn clinical practice guidelines in oncology. J Natl Compr Canc Netw. (2021) 19:839–68. doi: 10.6004/jnccn.2021.0032 34340212

[2] Jing Y Hu J Luo R Mao Y Luo Z Zhang M. Prevalence and characteristics of adrenal tumors in an unselected screening population : A cross-sectional study. Ann Intern Med. (2022) 175:1383–91. doi: 10.7326/M22-1619 36095315

[3] Soon PS McDonald KL Robinson BG Sidhu SB. Molecular markers and the pathogenesis of adrenocortical cancer. Oncologist. (2008) 13:548–61. doi: 10.1634/theoncologist.2007-0243 18515740

[4] Sidhu S Martin E Gicquel C Melki J Clark SJ Campbell P. Mutation and methylation analysis of tp53 in adrenal carcinogenesis. Eur J Surg Oncol. (2005) 31:549–54. doi: 10.1016/j.ejso.2005.01.013 15922892

[5] Gicquel C Bertagna X Gaston V Coste J Louvel A Baudin E. Molecular markers and long-term recurrences in a large cohort of patients with sporadic adrenocortical tumors. Cancer Res. (2001) 61:6762–7. 11559548

[6] Pinto EM Chen X Easton J Finkelstein D Liu Z Pounds S. Genomic landscape of pediatric adrenocortical tumors. Nat Commun. (2015) 6:6302. doi: 10.1038/ncomms7302 25743702 PMC4352712

[7] Scicluna P Caramuta S Kjellin H Xu C Frobom R Akhtar M. Altered expression of the igf2−H19 locus and mitochondrial respiratory complexes in adrenocortical carcinoma. Int J Oncol. (2022) 61:140. doi: 10.3892/ijo.2022.5430 36169175 PMC9529429

[8] Bock FJ Tait SWG. Mitochondria as multifaceted regulators of cell death. Nat Rev Mol Cell Biol. (2020) 21:85–100. doi: 10.1038/s41580-019-0173-8 31636403

[9] Luo Y Ma J Lu W. The significance of mitochondrial dysfunction in cancer. Int J Mol Sci. (2020) 21:5598. doi: 10.3390/ijms21165598 32764295 PMC7460667

[10] Song J Herrmann JM Becker T. Quality control of the mitochondrial proteome. Nat Rev Mol Cell Biol. (2021) 22:54–70. doi: 10.1038/s41580-020-00300-2 33093673

[11] Kang R Xie Y Zeh HJ Klionsky DJ Tang D. Mitochondrial quality control mediated by pink1 and prkn: links to iron metabolism and tumor immunity. Autophagy. (2019) 15:172–3. doi: 10.1080/15548627.2018.1526611 PMC6287677 30252570

[12] 2Li W He P Huang Y Li YF Lu J Li M. Selective autophagy of intracellular organelles: recent research advances. Theranostics. (2021) 11:222–56. doi: 10.7150/thno.49860 PMC7681076 33391472

[13] Cai C Wu F He J Zhang Y Shi N Peng X. Mitochondrial quality control in diabetic cardiomyopathy: from molecular mechanisms to therapeutic strategies. Int J Biol Sci. (2022) 18:5276–90. doi: 10.7150/ijbs.75402 PMC9461654 36147470

[14] Chang X Toan S Li R Zhou H. Therapeutic strategies in ischemic cardiomyopathy: focus on mitochondrial quality surveillance. EBioMedicine. (2022) 84:104260. doi: 10.1016/j.ebiom.2022.104260 36122552 PMC9490489

[15] Picca A Mankowski RT Burman JL Donisi L Kim JS Marzetti E. Mitochondrial quality control mechanisms as molecular targets in cardiac ageing. Nat Rev Cardiol. (2018) 15:543–54. doi: 10.1038/s41569-018-0059-z PMC6283278 30042431

[16] Zhong FM Yao FY Liu J Li MY Jiang JY Cheng Y. Splicing factor-mediated regulation patterns reveals biological characteristics and aid in predicting prognosis in acute myeloid leukemia. J Transl Med. (2023) 21:6. doi: 10.1186/s12967-022-03868-9 36611187 PMC9824960

[17] Zou R Shi W Qiu J Zhou N Du N Zhou H. Empagliflozin attenuates cardiac microvascular ischemia/reperfusion injury through improving mitochondrial homeostasis. Cardiovasc Diabetol. (2022) 21:106. doi: 10.1186/s12933-022-01532-6 35705980 PMC9202214

[18] Rath S Sharma R Gupta R Ast T Chan C Durham TJ. Mitocarta3.0: an updated mitochondrial proteome now with sub-organelle localization and pathway annotations. Nucleic Acids Res. (2021) 49:D1541–D7. doi: 10.1093/nar/gkaa1011 PMC7778944 33174596

[19] Mariathasan S Turley SJ Nickles D Castiglioni A Yuen K Wang Y. Tgfbeta attenuates tumor response to pd-L1 blockade by contributing to exclusion of T cells. Nature. (2018) 554:544–8. doi: 10.1038/nature25501 PMC6028240 29443960

[20] Thorsson V Gibbs DL Brown SD Wolf D Bortone DS Ou Yang TH. The immune landscape of cancer. Immunity. (2018) 48:812–30 e14. doi: 10.1016/j.immuni.2018.03.023 29628290 PMC5982584

[21] Vara-Perez M Felipe-Abrio B Agostinis P. Mitophagy in cancer: A tale of adaptation. Cells. (2019) 8:493. doi: 10.3390/cells8050493 31121959 PMC6562743

[22] Wang J Zhou H. Mitochondrial quality control mechanisms as molecular targets in cardiac ischemia-reperfusion injury. Acta Pharm Sin B. (2020) 10:1866–79. doi: 10.1016/j.apsb.2020.03.004 PMC7606115 33163341

[23] Tang Y Wang L Qin J Lu Y Shen HM Chen HB. Targeting mitophagy to promote apoptosis is a potential therapeutic strategy for cancer. Autophagy. (2023) 19:1031–3. doi: 10.1080/15548627.2022.2112830 PMC9980546 35968729

[24] Panigrahi DP Praharaj PP Bhol CS Mahapatra KK Patra S Behera BP. The emerging, multifaceted role of mitophagy in cancer and cancer therapeutics. Semin Cancer Biol. (2020) 66:45–58. doi: 10.1016/j.semcancer.2019.07.015 31351198

